# Supplementary material for: Protocol for LAsting Symptoms after Oesophageal Resectional Surgery (LASORS): multicentre validation cohort study
Source: BMJ Open. 2020 Jun 3;10(6):e034897. doi: 10.1136/bmjopen-2019-034897 (PMC7279661; doi:10.1136/bmjopen-2019-034897)
Supplement: Supplementary data [file bmjopen-2019-034897supp001.pdf]

Imperial College London

Division of Surgery
Department of Surgery and Cancer
Imperial College London
Biodynamics laboratory, 7th Floor
Charing Cross Hospital
Fulham Palace Road
London W6 8RF
Tel: +44 (0) 203 312 2125
s.markar@imperial.ac.uk
www.imperial.ac.uk

Name of Researchers: Dr Sheraz R. Markar, Professor George B. Hanna, Professor Tim Underwood, Mr Ewen Griffiths, Mr Ravi Vohra, Mr James Gossage, Mr Stuart Mercer, Mr Bilal Alkhaffaf, Mr Arun Ariyarathenam, Mr Shameen Jahoo, Mr John Findlay

Participant Identification Number for this trial:

CONSENT FORM

LAsting Symptoms after Oesophageal Resectional Surgery (LASORS)

1. I confirm that I have read and understood the Patient Information Sheet dated 25.03.19 (version 1) for the above study. I have had the opportunity to consider the information, ask questions and have had these answered satisfactorily. ☐
2. I understand that my participation is voluntary and that I am free to withdraw at any time without giving a reason, and without my medical care, education, employment or legal rights being affected. ☐
3. I agree to my data, including personal identifiable data, being stored on Imperial College London computer system and understand my research data will be stored for 10 years following completion of the study. ☐
4. I understand that sections of my research notes and/ data may be accessed by responsible persons from the research team, from Imperial College London and/or Imperial College Healthcare NHS Trust or from regulatory authorities where it is relevant to the research and in order to check that it is being conducted correctly. ☐
5. I agree for my contact details to be stored on Imperial College London Secure Database in the event that I may request for a research summary. Once the research summary has been sent out, I understand my personal contact details will be deleted. ☐
6. I agree to take part in the above study. ☐

Full name of Participant	Date	Signature
Name of person taking consent	Date	Signature

Consent Form: Version 1.0 Date 25.03.2019
IRAS No: XXX
1 copy to the patient, 1 copy to site file, 1copy to the patient file
