# Supplementary material for: Protocol for LAsting Symptoms after Oesophageal Resectional Surgery (LASORS): multicentre validation cohort study
Source: BMJ Open. 2020 Jun 3;10(6):e034897. doi: 10.1136/bmjopen-2019-034897 (PMC7279661; doi:10.1136/bmjopen-2019-034897)
Supplement: Supplementary data [file bmjopen-2019-034897supp002.pdf]

Division of Surgery
Department of Surgery and Cancer
Imperial College London
Biodynamics laboratory, 7th Floor
Charing Cross Hospital
Fulham Palace Road
London W6 8RF
Tel: +44 (0) 203 312 2125
s.markar@imperial.ac.uk
www.imperial.ac.uk

Name of Researchers: Dr Sheraz R. Markar, Professor George B. Hanna, Professor Tim Underwood, Mr Ewen Griffiths, Mr Ravi Vohra, Mr James Gossage, Mr Stuart Mercer, Mr Bilal Alkhaffaf, Mr Arun Ariyarathenam, Mr Shameen Jahoo, Mr John Findlay

CASE REPORT FORM

LAsting Symptoms after Oesophageal Resectional Surgery (LASORS)

Participant Identification Number for this trial: ....

Name of place of surgery: …………………………………………………………………………

Current age of patient: ……………………………………………………………………….....

Gender: Male ❍

Female ❍

Height at surgery (cm): ………………………………………………………………………..

Weight at surgery (kg): ………………………………………………………………………..

Current weight (kg): ……………………………………………………………………….

Date of surgery: ………………………………………………………………………………….

Preoperative medical comorbidties:

- Ischaemic Heart Disease ❍
- Congestive heart failure ❍
- Cerebrovascular disease ❍
- Peripheral Vascular disease ❍
- Dementia ❍
- Chronic obstructive pulmonary disease ❍
- Peptic ulcer disease ❍
- Mild liver disease ❍
- Diabetes ❍
- Renal disease ❍

ASA Grade:

- I ❍
- II ❍
- III ❍
- IV ❍

Case Report Form: Version 1.0 Date 25.03.2019
IRAS No: xxx

Anastomosis location:
- Cervical ❍
- Thoracic ❍

Surgical technique:
- Transhiatal ❍
- Ivor-Lewis (2-stage) ❍
- McKeown (3-stage) ❍
- Left thoracoabdominal ❍
- Colonic interposition ❍
- Other – please specify ❍

[ ]

How was the anastomosis constructed?
- Linear Stapler ❍
- Circular Stapler (please state diameter if known ❍
- Hand sewn ❍

Alignment of anastomosis:
- End to end ❍
- End to side ❍
- Side to side ❍
- Other (please specify) ❍

[ ]

Surgical access:
- Total Minimally Invasive Oesophagectomy ❍
- Hybrid Minimally Invasive Oesophagectomy (Open abdominal and thoracoscopy) ❍
- Hybrid Minimally Invasive Oesophagectomy (Laparoscopy and open thoracotomy) ❍
- Total Open Oesophagectomy ❍

Pyloric management:
- No drainage procedure ❍
- Pyloroplasty ❍
- Pyloromyotomy ❍
- Manual pyloric dilatation or stretch ❍
- Botulinum injection ❍
- Other (please specify) ❍

[ ]

Case Report Form: Version 1.0 Date 25.03.2019
IRAS No: xxx

Was a feeding jejunostomy inserted? Yes ❍

No ❍

Neoadjuvant therapy: Combination chemoradiotherapy ❍

Radiotherapy alone ❍

Chemotherapy alone ❍

None ❍

Chemotherapy or chemoradiotherapy regimen:

CROSS ❍

MAGIC ❍

FLOT ❍

Other ❍

Unknown ❍

CROSS - Paclitaxel (50 mg/m2) and Carboplatin (Area-Under-Curve = 2) administered by i.v. infusion on days 1, 8, 15, 22, and 29. External beam radiation with a total dose of 41.4 Gy given in 23 fractions of 1.8 Gy, 5 fractions a week.

MAGIC - Three preoperative and three postoperative cycles of intravenous epirubicin (50 mg per square meter of body-surface area) and cisplatin (60 mg per square meter) on day 1, and a continuous intravenous infusion of fluorouracil (200 mg per square meter per day) for 21 days.

Adjuvant therapy: Combination chemoradiotherapy ❍

Radiotherapy alone ❍

Chemotherapy alone ❍

None ❍

Pathological T stage: T0 ❍

T1 ❍

T2 ❍

T3 ❍

T4 ❍

Pathological N stage 7th Edition: N0 ❍

N1 ❍

N2+ ❍

Pathological TNM stage 7th Edition: Stage I ❍

Stage II ❍

Stage III ❍

Stage IV ❍

Case Report Form: Version 1.0 Date 25.03.2019
IRAS No: xxx

Tumour histological subtype: Adenocarcinoma ❍
SCC ❍
Other ❍

Postoperative complications (use complications platform from Low DE et al. Annals of Surgery 262 (2):286-294 2015):

- Pulmonary ❍
- Cardiac ❍
- Gastrointestinal ❍
- Urologic ❍
- Thromboembolic ❍
- Neurologic/psychiatric ❍
- Infection ❍
- Wound/diaphragm ❍
- Other ❍

Please specify pulmonary complication:

- Pneumonia ❍
- Pleural effusion requiring additional drainage procedure ❍
- Pneumothorax requiring treatment ❍
- Atelectasis mucous plugging requiring bronchoscopy ❍
- Respiratory failure requiring reintubation ❍
- Acute respiratory distress syndrome (Berlin definition) ❍
- Acute aspiration ❍
- Tracheobronchial injury ❍
- Chest tube maintenance for air leak for >10 days postoperatively ❍

Please specify cardiac complication:

- Cardiac arrest requiring CPR ❍
- Myocardial infarction as defined by the World Health Organisation ❍
- Dysrhythmia atrial requiring treatment ❍
- Dysrhythmia ventricular requiring treatment ❍
- Congestive heart failure requiring treatment ❍
- Pericarditis requiring treatment ❍

Please specify gastrointestinal complication:

- Oesophagoenteric leak from anastomosis, stapled line or localised conduit necrosis ❍
- Conduit necrosis/failure ❍
- Ileus defined as small bowel dysfunction preventing or delayed enteral feeding ❍
- Small bowel obstruction ❍
- Feeding J-tube complication ❍

Case Report Form: Version 1.0 Date 25.03.2019
IRAS No: xxx

Pyloromyotomy/pyloroplasty complication ❍

Clostridium Difficile infection ❍

Gastrointestinal bleeding requiring intervention or transfusion ❍

Delayed conduit emptying requiring intervention or delayed discharge or requiring maintenance of nasogastric drainage >7 days postoperatively ❍

Pancreatitis ❍

Liver dysfunction ❍

Please specify urological complication:

Acute renal insufficiency (defined as doubling of baseline creatinine) ❍

Acute renal failure requiring dialysis ❍

Urinary tract infection ❍

Urinary retention requiring reinsertion of urinary catheter, delaying discharge or discharge with a urinary catheter ❍

Please specify thromboembolic complication:

Deep vein thrombosis ❍

Pulmonary embolus ❍

Stroke (CVA) ❍

Peripheral thrombophlebitis ❍

Please specify neurologic/psychiatric complication:

Recurrent nerve injury ❍

Other neurologic injury ❍

Acute delirium ❍

Delirium tremens ❍

Please specify infection complication:

Wound infection requiring opening of wound or antibiotics ❍

Central i.v. line infection requiring removal of antibiotics ❍

Intrathoracic/intra-abdominal abscess ❍

Generalised sepsis ❍

Other infections requiring antibiotics ❍

Please specify wound/diaphragm complication:

Thoracic wound dehiscence ❍

Acute abdominal wall dehiscence/hernia ❍

Acute diaphragmatic hernia ❍

Please specify other complication:

Case Report Form: Version 1.0 Date 25.03.2019
IRAS No: xxx

Chyle leak ❍

Reoperation for reasons other than bleeding, anastomotic leak or conduit necrosis ❍

Multiple organ dysfunction syndrome ❍

Late symptoms requiring intervention (>90 days following surgery):

Stricture ❍

Delaying emptying (requiring dilatation) ❍

Other ❍

[ ]

Maximum Clavien-Dindo Classification of postoperative complication (include 30-day complications or in-patient stay – whichever is the longest)

I ❍

II ❍

III ❍

IV ❍

Case Report Form: Version 1.0 Date 25.03.2019
IRAS No: xxx
