# Supplementary material for: Protocol for LAsting Symptoms after Oesophageal Resectional Surgery (LASORS): multicentre validation cohort study
Source: BMJ Open. 2020 Jun 3;10(6):e034897. doi: 10.1136/bmjopen-2019-034897 (PMC7279661; doi:10.1136/bmjopen-2019-034897)
Supplement: Supplementary data [file bmjopen-2019-034897supp003.pdf]

LASORS tool - Satisfaction

INSTRUCTIONS:

- Please try to answer all questions.
- Circle the answer that you believe describes you best.

1	2	3	4	5	6	7
Disagree Completely			Neutral			Agree Completely

1. LASOR questionnaire was easy to complete.	1	2	3	4	5	6	7
2. LASOR questionnaire was understandable.	1	2	3	4	5	6	7
3. LASOR questionnaire did not take much time to complete.	1	2	3	4	5	6	7
4. LASOR questionnaire included the main symptoms I experience.	1	2	3	4	5	6	7
5. I preferred the LASOR questionnaire to the EORTC questionnaires	1	2	3	4	5	6	7
6. I would recommend using the LASOR questionnaire to other oesophageal cancer survivors.	1	2	3	4	5	6	7
