# Supplementary material for: Protocol for LAsting Symptoms after Oesophageal Resectional Surgery (LASORS): multicentre validation cohort study
Source: BMJ Open. 2020 Jun 3;10(6):e034897. doi: 10.1136/bmjopen-2019-034897 (PMC7279661; doi:10.1136/bmjopen-2019-034897)
Supplement: Author's manuscript [file bmjopen-2019-034897.draft_revisions.pdf]

BMJ Open is committed to open peer review. As part of this commitment we make the peer review history of every article we publish publicly available.

When an article is published we post the peer reviewers' comments and the authors' responses online. We also post the versions of the paper that were used during peer review. These are the versions that the peer review comments apply to.

The versions of the paper that follow are the versions that were submitted during the peer review process. They are not the versions of record or the final published versions. They should not be cited or distributed as the published version of this manuscript.

BMJ Open is an open access journal and the full, final, typeset and author-corrected version of record of the manuscript is available on our site with no access controls, subscription charges or pay-per-view fees (http://bmjopen.bmj.com).

If you have any questions on BMJ Open's open peer review process please email info.bmjopen@bmj.com

BMJ Open

LAsting Symptoms after Oesophageal Resectional Surgery (LASORS)

Journal:	*BMJ Open*
Manuscript ID	bmjopen-2019-034897
Article Type:	Protocol
Date Submitted by the Author:	17-Nov-2019

Complete List of Authors:	Markar, Sheraz; Imperial College London,<br>Griffiths, Ewen; University Hospitals Birmingham NHS Foundation Trust, Department of Upper GI Surgery<br>Behrens, Paul; University of Edinburgh<br>Singh, Pritam; Nottingham University Hospitals NHS Trust<br>Vohra, RS; Nottingham University Hospitals NHS Trust,<br>Gossage, James; St. Thomas' Hospital, London, Department of Surgery<br>Underwood, Tim; University Hospital Southampton, Division of Surgery, University Hospital Southampton NHS Foundation Trust, Tremona Road, Southampton, Hampshire, SO16 6YD<br>Hanna, George; Imperial College, Faculty of Medicine, Department of Surgery & Cancer

Keywords:	SURGERY, Oesophageal disease < GASTROENTEROLOGY, ONCOLOGY

SCHOLARONE™
Manuscripts

I, the Submitting Author has the right to grant and does grant on behalf of all authors of the Work (as defined in the below author licence), an exclusive licence and/or a non-exclusive licence for contributions from authors who are: i) UK Crown employees; ii) where BMJ has agreed a CC-BY licence shall apply, and/or iii) in accordance with the terms applicable for US Federal Government officers or employees acting as part of their official duties; on a worldwide, perpetual, irrevocable, royalty-free basis to BMJ Publishing Group Ltd ("BMJ") its licensees and where the relevant Journal is co-owned by BMJ to the co-owners of the Journal, to publish the Work in this journal and any other BMJ products and to exploit all rights, as set out in our licence.

The Submitting Author accepts and understands that any supply made under these terms is made by BMJ to the Submitting Author unless you are acting as an employee on behalf of your employer or a postgraduate student of an affiliated institution which is paying any applicable article publishing charge ("APC") for Open Access articles. Where the Submitting Author wishes to make the Work available on an Open Access basis (and intends to pay the relevant APC), the terms of reuse of such Open Access shall be governed by a Creative Commons licence – details of these licences and which Creative Commons licence will apply to this Work are set out in our licence referred to above.

Other than as permitted in any relevant BMJ Author's Self Archiving Policies, I confirm this Work has not been accepted for publication elsewhere, is not being considered for publication elsewhere and does not duplicate material already published. I confirm all authors consent to publication of this Work and authorise the granting of this licence.

For peer review only

Title: LAsting Symptoms after Oesophageal Resectional Surgery (LASORS)

Investigator(s): Sheraz R. Markar[1], Ewen A. Griffiths[2], Paul Behrens[3], Pritam Singh[4], Ravi Vohra[4], James Gossage[5], Tim Underwood[6], George B. Hanna[1], On behalf of the LASORS study group.

1 – Department of Surgery & Cancer, Imperial College London, UK.

2 – Department of Upper Gastrointestinal Surgery, University Hospitals Birmingham NHS Foundation Trust, Birmingham, UK.

3 – Patient representative, Reader in Law, University of Edinburgh, UK.

4 – Trent Oesophago-Gastric Unit, City Hospital Campus, Nottingham University Hospitals NHS Trust, Nottingham UK.

5 – Department of Upper GI Surgery, Guy's & St Thomas' Hospital, London UK.

6 – University of Southampton, UK.

Address for Correspondence: Sheraz R. Markar, NIHR Clinical Lecturer, Division of Surgery, Department of Surgery & Cancer, Imperial College London, 10th Floor QEQM Building, St Mary's Hospital, South Wharf Road, London, W2 1NY, UK

E-mail: s.markar@imperial.ac.uk

Telephone No: +44 (0)207 886 2125

Fax No: +44 (0)207 8862125

Article category: Protocol

Funding: Sheraz R. Markar is supported by the National Institute for Health Research (NIHR). Timothy J. Underwood is supported by a Cancer Research UK (CRUK) Advanced Clinician Scientist Fellowship.

Conflicts of Interests: The authors declared no conflict of interest

Word Count: 1093

For peer review only

Figures: 0 Tables: 2

Appendix: 0

For peer review only

ABSTRACT

Introduction

Surgery is the primary curative treatment for oesophageal cancer, with considerable recent improvements in long-term survival. However surgery has a long-lasting impact upon patient's health-related quality of life (HRQOL). Through a multi-centre European study, our research group was able to identify key symptoms that affect patient's HRQOL. These symptoms were combined to produce a tool to identify poor HRQOL following oesophagectomy (LASOR tool).

The objective of this multi-centre study is to validate a six-symptom clinical tool to identify patients with poor HRQOL for use in everyday clinical practice.

Methods and Analysis

Included patients will; (i) be aged 18 years or older, (ii) have undergone an oesophagectomy for cancer between 2015 and 2018, (iii) be at least 12-months after the completion of adjuvant oncological treatments. Patients will be given the previously created LASOR questionnaire. Each symptom from the LASOR questionnaire will be graded according to impact upon quality of life and frequency of the symptom, with a composite score from 0 to 5. The previously developed LASOR symptom tool will be validated against HRQOL as measured by the EORTC QLQC30 and OG25.

Sample size

With a predicted prevalence of poor HRQOL of 45%, based upon the previously generated LASOR clinical symptom tool, to validate this tool with a sensitivity and specificity of 80% respectively, a minimum of 640 patients will need to be recruited to the study.

For peer review only

Ethics & Dissemination

NHS Health Research Authority **(**North East – York Research Ethics Committee) approval was gained 8th November 2019 (REC reference 19/NE/0352). Multiple platforms will be used for the dissemination of the research data, including international clinical and patient group presentations and publication of research outputs in a high impact clinical journal.

Word count: 279

For peer review only

Article Summary

Strengths and limitations of this study

Strengths

- Large, multi-centre, contemporenous, European study of Quality of life after esophagectomy
- Comparison will be performed with more comprehensive quality of life tools (EORTC-QLQC30 and OG25)
- Quality of Life Questionnaire / LASOR tool has been developed in association with oesophageal cancer patient support group charities (Heartburn Cancer UK and Oesophageal Patients Association)

Limitations

- The LASOR tool will be validated in patients who have had potentially curative esophageal surgery within 1 – 4 years and could suffer some bias of well-motivated patients responding to invites and some patients suffering from symptoms of undiagnosed recurrent cancer being included.
- Patients who have already suffered recurrent cancer or early post-operative death will not be included.

For peer review only

Introduction

Globally, oesophageal cancer is the 12th most common cancer type and the 7th most common cause of cancer related death, with an overall five-year survival of less than 20% [1–3]. The mainstay of curative treatment for oesophageal cancer is surgical resection, which is often combined with chemo- or chemoradiotherapy [4,5]. This multimodality approach to treatment, along-with centralisation of oesophageal cancer surgery to high volume centers and the introduction of minimally invasive approaches to surgery, have been associated with major recent improvements in short-term and oncological outcomes and survival [6,7,8].

With the recent improvements in survival, the assessment of patient reported outcome measures including, patient's health-related quality of life (HRQOL) and the impact of long-term symptoms in survivorship has become increasingly important. However, current data regarding the long-term symptom burden following potentially curative oesophagectomy are limited and evidence-based interventions are lacking. One population-based cohort study suggested approximately 40% of patients seek medical attention for long-term symptoms, associated with increased depression and anxiety [9].

The European Organisation for Research and Treatment of Cancer (EORTC) have developed questionnaires for the assessment of HRQOL during treatment for esophageal cancer [10,11]. These tools have been widely used in a research setting to assess the impact on HRQOL of multimodality treatment, variations in surgical technique and complications [12,13]. However, the EORTC-QLQC30 and OG25 modules, are often considered too cumbersome to be routinely used in clinical practice. Furthermore, these questionnaires are not designed for use in cancer-free

For peer review only

survivorship, when the nature of symptoms and their HRQOL impact may be significantly different, compared with the context of diagnosis and treatment.

Through a multi-center European study of 876 patients, we recently identified three key symptoms that were independently associated with poor HRQOL as measured by validated EORTC tools [14]. We presented these findings to the Oesophageal patient association (UK) and Heartburn Cancer UK patient support groups who identified a further three symptoms to be included in the final clinical symptom tool that we aim to validate in this present UK study.

For peer review only

Objectives

Global objective:

- To validate a six-symptom clinical tool to identify patients with poor HRQOL for use in everyday clinical practice.

Specific objectives:

1. Validate a six symptom clinical tool to identify patients with poor HRQOL as measured by current EORTC QLQC30 and OG25 tools.
2. To assess patient acceptability of this clinical symptom-based tool for postoperative clinical follow-up.

For peer review only

Methods and analysis

Study Design

Cross-sectional study to validate a symptom questionnaire in the identification of patients with poor HRQOL following oesophageal cancer surgery. Eligible patients from the United Kingdom will be invited to complete the LASOR tool (Table 1) and the EORTC QLQC30 and OG25 questionnaires.

Measures:

Archival data

Demographic information: Data on pre-treatment; patient age, sex, body mass index, ethnic background, socioeconomic status (Carstairs index), education level, smoking status, and medical comorbidities will be collected through review of medical records.

Tumour and treatment information: Information on stage, tumour location, type of surgery, postoperative complications as defined by the Esophageal Complications Consensus Group (ECCG) [15], and neoadjuvant and adjuvant therapy used will be collected by reviewing medical records.

Complications after 12months: Specific data on long-term complications including anastomotic stricture requiring dilatation and hiatal hernia, will be collected by reviewing medical records.

Outcome data

Each symptom from the LASOR questionnaire will be graded according to impact upon quality of life and frequency of the symptom, with a composite score from 0 to 5. Each EORTC HROQL symptom item comprised 4 categories on a Likert scale: 1, not at all; 2, a little; 3, quite a bit; 4, very much. The previously developed LASOR

For peer review only

symptom tool will be validated against HRQOL as measured by the EORTC QLQC30 and OG25.

Inclusion criteria

- Patients aged over 18 years at the time of surgery.
- Patients treated with oesophagectomy for oesophageal or gastro-oesophageal junctional cancer between January 2015 and June 2018.
- Patients at least 12 months post completion of cancer treatment (surgical or oncological)

Exclusion Criteria

- Any patient who lacks capacity or is unable to provide informed consent.
- Any patient below 18 years of age at the time of surgery.
- Any patient with evidence of cancer recurrence

Sample size: With a predicted prevalence of poor HRQOL of 45%, based upon the previously generated LASOR clinical symptom tool, to validate this tool with a sensitivity and specificity of 80% respectively, a minimum of 640 patients would need to be recruited to the study.

Statistical methodology:

Each symptom from the LASOR questionnaire will graded according to impact upon quality of life and frequency of the symptom, with a composite score from 0 to 5 (Table 2). Each EORTC HROQL symptom item comprises 4 categories on a Likert scale: 1, not at all; 2, a little; 3, quite a bit; 4, very much. Linear transformation of Likert scores for answers in each conceptual area will be performed as per EORTC recommendations. Symptom scores hence comprise a numeric value from 0 to 100, with higher scores indicating more pervasive symptoms. Poor HRQOL will be defined

For peer review only

as having poor function and poor symptom in QLQ-C30 and QlQ-OG25 (by answering “Quite a Bit”/”Very Much” problems to at least one question each in function and symptom scales). Patients who do not answer “Quite a Bit”/”Very Much” will be considered as having good HRQOL. The area under the receiver operating characteristics curve (AUC) will be utilized as a measure of overall accuracy of the prediction model from the LASOR tool in the identification of patients with poor HRQOL as measured by EORTC QLQ-C30 and QlQ-OG25 tools.

For peer review only

Dissemination

Multiple methods of dissemination will be employed to ensure the findings from this research will reach relevant stakeholders including patients, primary care practitioners, scientists, hospital specialists in gastroenterology, oncology and surgery, health policy makers and commissioners as well as healthcare regulatory bodies. The study findings will be presented at international gastroenterology, oncology and surgical research meetings. The findings of this research will also be presented to relevant patient groups. Ultimately, we plan to publish the results of this research in a high impact factor clinical journal to allow widespread dissemination of this research.

Ethics

NHS Health Research Authority (North East – York Research Ethics Committee) approval was gained 8th November 2019 (REC reference 19/NE/0352).

Author contributions

SR Markar; Study conception, design, and drafting of full protocol.

EA Griffiths; study design and revising full protocol.

P Singh; study design and revising full protocol.

R Vohra; study design and revising full protocol.

J Gossage; study design and revising full protocol.

TJ Underwood; study design and revising full protocol.

For peer review only

Funding Statement

No funding has been gained for this study at present.

Competing interests

None

Collaborating authors

On behalf of the LASORS study group.

For peer review only

References

1. Fitzmaurice C, Akinyemiju TF, Al Lami FH, et al. Global, regional, national cancer incidence, mortality, years of life lost, years lived with disability, and disability-adjusted life-years for 29 cancer groups, 1990 to 2016: a systematic analysis for the global burden of disease study. JAMA Oncol 2018; 4: 1553 – 68.

2. Siegel RL, Miller KD, Jemal A. Cancer statistics 2018. CA Cancer J Clin 2018; 68: 7–30.

3. Anderson LA, Tavilla A, Brenner H, et al. Survival for oesophageal, stomach and small intestine cancers in Europe 1999–2007: Results from EUROCARE-5. Eur J Cancer 2015; 51: 2144–57.

4. Shapiro J, Van Lanschot JJB, Hulshof MCCM, et al. Neoadjuvant chemoradiotherapy plus surgery versus surgery alone for oesophageal or junctional (CROSS): long-term results of a randomised controlled trial. Lancet Oncol 2015; 16: 1090–8.

5. Alderson D, Cunningham D, Nankivell M, et al. Neoadjuvant cisplatin and fluorouracil versus epirubicin, cisplatin, and capecitabine followed by resection in patients with esophageal adenocarcinoma (UK MRCOE05): an open-label, randomised phase 3 trial. Lancet Oncol 2017; 18: 1249 – 60.

6. Finks JF, Osborne NH, Birkmeyer JD. Trends in hospital volume and operative mortality for high-risk surgery. N Engl J Med 2011; 364: 2128–37.

7. Palser TR, Cromwell DA, Hardwick RH, et al. Re-organisation of oesophago-gastric cancer care in England: progress and remaining challenges. BMC Health Serv Res. 2009; 9:204.

For peer review only

8. Mariette C, Markar SR, Dabakuyo-Yonli TS et al. Hybrid minimally invasive esophagectomy for esophageal cancer. N Engl J Med 2019; 380: 152–62.

9. Bouras G, Markar SR, Burns EM, et al. The psychological impact of symptoms related to esophagogastric cancer resection presenting in primary care: a national linked database study. Eur J Surg Oncol 2017; 43: 454–60.

10. McKernan M, McMillan DC, Anderson JR, et al. The relationship between quality of life (EORTC QLQ-C30) and survival in patients with gastro-oesophageal cancer. Br J Cancer 2008; 98: 888–93.

11. Lagergren P, Fayers P, Conroy T, et al. Clinical and psychometric validation of a questionnaire module, the EORTC QLQ-O25, to assess health-related quality of life in patients with cancer of the oesophagus, the oesophago-gastric junction and the stomach. Eur J Cancer 2007; 43: 2066–73.

12. Noordman BJ, Verdam MGE, Lagarde SM, et al. Effect of neoadjuvant chemoradiotherapy on health-related quality of life in esophageal or junctional cancer: results from the randomized CROSS trial. J Clin Oncol 2018; 36: 268–75.

13. Kauppila JH, Johar A, Lagergren P. Medical and surgical complications and health-related quality of life after esophageal cancer surgery. Ann Surg 2018 [Epub ahead of print].

14. Markar SR, Zaninotto G, Castoro C, et al. LAsting Symptoms after Esophageal Resection (LASER) – European Multi-center Cross-sectional Study. *Presented at International Society for Diseases of Esophagus meeting Vienna 2018.*

15. Low DE, Alderson D, Cecconello I, et al. International consensus on standardization of data collection for complications associated with esophagectomy: esophagectomy complications consensus group (ECCG). Ann Surg 2015; 262: 286–94.

For peer review only

	Q1. Do you have any of the following symptoms and how often? Please mark						Q2. What is the impact of these symptoms to your quality of life?		
**Symptom**	**Never**	**Rarely**	**Weekly**	**Daily**	**Multiple times per day**		**None**	**Some**	**Substantial**
Low mood									
Reduced energy or activity tolerance									
Pain on scars from chest									
Heartburn/acid/bile (sour/bitter tasting)									
Diarrhoea (>3 times per day) unrelated to eating									
Bloating or cramping after eating									

Table 1: LASOR symptom tool

For peer review only

Symptom Level	QOL impact and Frequency
0	No symptom present
1	QOL impact = None
2	QOL impact = Some & Frequency = rarely / weekly
3	QOL impact = Some & Frequency = daily / multiple
4	QOL impact = Substantial & Frequency = rarely / weekly
5	QOL impact = Substantial & Frequency = daily / multiple

Table 2: Symptom based grading system including prevalence and impact on Quality of life (QOL)

BMJ Open

LAsting Symptoms after Oesophageal Resectional Surgery (LASORS)

Journal:	*BMJ Open*
Manuscript ID	bmjopen-2019-034897.R1
Article Type:	Protocol
Date Submitted by the Author:	03-Feb-2020
Complete List of Authors:	Markar, Sheraz; Imperial College London,<br>Griffiths, Ewen; University Hospitals Birmingham NHS Foundation Trust, Department of Upper GI Surgery<br>Behrens, Paul; University of Edinburgh<br>Singh, Pritam; Nottingham University Hospitals NHS Trust<br>Vohra, RS; Nottingham University Hospitals NHS Trust,<br>Gossage, James; St. Thomas' Hospital, London, Department of Surgery<br>Underwood, Tim; University Hospital Southampton, Division of Surgery, University Hospital Southampton NHS Foundation Trust, Tremona Road, Southampton, Hampshire, SO16 6YD<br>Hanna, George; Imperial College, Faculty of Medicine, Department of Surgery & Cancer
**Primary Subject Heading**:	Surgery
Secondary Subject Heading:	Oncology, Rehabilitation medicine, Patient-centred medicine
Keywords:	SURGERY, Oesophageal disease < GASTROENTEROLOGY, ONCOLOGY

SCHOLARONE™
Manuscripts

I, the Submitting Author has the right to grant and does grant on behalf of all authors of the Work (as defined in the below author licence), an exclusive licence and/or a non-exclusive licence for contributions from authors who are: i) UK Crown employees; ii) where BMJ has agreed a CC-BY licence shall apply, and/or iii) in accordance with the terms applicable for US Federal Government officers or employees acting as part of their official duties; on a worldwide, perpetual, irrevocable, royalty-free basis to BMJ Publishing Group Ltd ("BMJ") its licensees and where the relevant Journal is co-owned by BMJ to the co-owners of the Journal, to publish the Work in this journal and any other BMJ products and to exploit all rights, as set out in our licence.

The Submitting Author accepts and understands that any supply made under these terms is made by BMJ to the Submitting Author unless you are acting as an employee on behalf of your employer or a postgraduate student of an affiliated institution which is paying any applicable article publishing charge ("APC") for Open Access articles. Where the Submitting Author wishes to make the Work available on an Open Access basis (and intends to pay the relevant APC), the terms of reuse of such Open Access shall be governed by a Creative Commons licence – details of these licences and which Creative Commons licence will apply to this Work are set out in our licence referred to above.

Other than as permitted in any relevant BMJ Author's Self Archiving Policies, I confirm this Work has not been accepted for publication elsewhere, is not being considered for publication elsewhere and does not duplicate material already published. I confirm all authors consent to publication of this Work and authorise the granting of this licence.

For peer review only

Title: LAsting Symptoms after Oesophageal Resectional Surgery (LASORS)

Investigator(s): Sheraz R. Markar[1], Ewen A. Griffiths[2], Paul Behrens[3], Pritam Singh[4], Ravi Vohra[4], James Gossage[5], Tim Underwood[6], George B. Hanna[1], On behalf of the LASORS study group.

1 – Department of Surgery & Cancer, Imperial College London, UK.

2 – Department of Upper Gastrointestinal Surgery, University Hospitals Birmingham NHS Foundation Trust, Birmingham, UK.

3 – Patient representative, Reader in Law, University of Edinburgh, UK.

4 – Trent Oesophago-Gastric Unit, City Hospital Campus, Nottingham University Hospitals NHS Trust, Nottingham UK.

5 – Department of Upper GI Surgery, Guy's & St Thomas' Hospital, London UK.

6 – University of Southampton, UK.

Address for Correspondence: Sheraz R. Markar, NIHR Clinical Lecturer, Division of Surgery, Department of Surgery & Cancer, Imperial College London, 10th Floor QEQM Building, St Mary's Hospital, South Wharf Road, London, W2 1NY, UK

E-mail: s.markar@imperial.ac.uk

Telephone No: +44 (0)207 886 2125

Fax No: +44 (0)207 8862125

Article category: Protocol

Funding: Sheraz R. Markar is supported by the National Institute for Health Research (NIHR). Timothy J. Underwood is supported by a Cancer Research UK (CRUK) Advanced Clinician Scientist Fellowship.

Conflicts of Interests: The authors declared no conflict of interest

Word Count: 1244 Figures: 0 Tables: 2 Appendix: 1

For peer review only

ABSTRACT

Introduction

Surgery is the primary curative treatment for oesophageal cancer, with considerable recent improvements in long-term survival. However surgery has a long-lasting impact upon patient's health-related quality of life (HRQOL). Through a multi-centre European study, our research group was able to identify key symptoms that affect patient's HRQOL. These symptoms were combined to produce a tool to identify poor HRQOL following oesophagectomy (LASOR tool).

The objective of this multi-centre study is to validate a six-symptom clinical tool to identify patients with poor HRQOL for use in everyday clinical practice.

Methods and Analysis

Included patients will; (i) be aged 18 years or older, (ii) have undergone an oesophagectomy for cancer between 2015 and 2018, (iii) be at least 12-months after the completion of adjuvant oncological treatments. Patients will be given the previously created LASOR questionnaire. Each symptom from the LASOR questionnaire will be graded according to impact upon quality of life and frequency of the symptom, with a composite score from 0 to 5. The previously developed LASOR symptom tool will be validated against HRQOL as measured by the EORTC QLQC30 and OG25.

Sample size

With a predicted prevalence of poor HRQOL of 45%, based upon the previously generated LASOR clinical symptom tool, to validate this tool with a sensitivity and specificity of 80% respectively, a minimum of 640 patients will need to be recruited to the study.

For peer review only

Ethics & Dissemination

NHS Health Research Authority **(**North East – York Research Ethics Committee) approval was gained 8th November 2019 (REC reference 19/NE/0352). Multiple platforms will be used for the dissemination of the research data, including international clinical and patient group presentations and publication of research outputs in a high impact clinical journal.

Word count: 279

For peer review only

Article Summary

Strengths and limitations of this study

Strengths

- Large, multi-centre, contemporenous, European study of Quality of life after esophagectomy
- Comparison will be performed with more comprehensive quality of life tools (EORTC-QLQC30 and OG25)
- Quality of Life Questionnaire / LASOR tool has been developed in association with oesophageal cancer patient support group charities (Heartburn Cancer UK and Oesophageal Patients Association)

Limitations

- The LASOR tool will be validated in patients who have had potentially curative esophageal surgery within 1 – 4 years and could suffer some bias of well-motivated patients responding to invites and some patients suffering from symptoms of undiagnosed recurrent cancer being included.
- Patients who have already suffered recurrent cancer or early post-operative death will not be included.

For peer review only

Introduction

Globally, oesophageal cancer is the 12th most common cancer type and the 7th most common cause of cancer related death, with an overall five-year survival of less than 20% [1–3]. The mainstay of curative treatment for oesophageal cancer is surgical resection, which is often combined with chemo- or chemoradiotherapy [4,5]. This multimodality approach to treatment, along-with centralisation of oesophageal cancer surgery to high volume centers and the introduction of minimally invasive approaches to surgery, have been associated with major recent improvements in short-term and oncological outcomes and survival [6,7,8].

With the recent improvements in survival, the assessment of patient reported outcome measures including, patient's health-related quality of life (HRQOL) and the impact of long-term symptoms in survivorship has become increasingly important. However, current data regarding the long-term symptom burden following potentially curative oesophagectomy are limited and evidence-based interventions are lacking. One population-based cohort study suggested approximately 40% of patients seek medical attention for long-term symptoms, associated with increased depression and anxiety [9].

The European Organisation for Research and Treatment of Cancer (EORTC) have developed questionnaires for the assessment of HRQOL during treatment for esophageal cancer [10,11]. These tools have been widely used in a research setting to assess the impact on HRQOL of multimodality treatment, variations in surgical technique and complications [12,13]. However, the EORTC-QLQC30 and OG25 modules, are often considered too cumbersome to be routinely used in clinical practice. Furthermore, despite frequently being used in cancer-free survivorship,

For peer review only

these questionnaires are not designed for this purpose, when the nature of symptoms and their HRQOL impact may be significantly different, compared with the context of diagnosis and treatment.

Through a multi-center European study of 876 patients, we recently identified three key symptoms that were independently associated with poor HRQOL as measured by validated EORTC tools [14]. We presented these findings to the Oesophageal patient association (UK) and Heartburn Cancer UK patient support groups who identified a further three symptoms to be included in the final clinical symptom tool that we aim to validate in this present UK study.

For peer review only

Objectives

Global objective:

- To validate a six-symptom clinical tool to identify patients with poor HRQOL more than one-year after surgery, for use in everyday clinical practice.

Specific objectives:

1. Validate a six symptom clinical tool to identify patients with poor HRQOL as measured by current EORTC QLQC30 and OG25 tools.
2. To assess patient acceptability of this clinical symptom-based tool for postoperative clinical follow-up.

For peer review only

Methods and analysis

Study Design

Cross-sectional study to validate a symptom questionnaire in the identification of patients with poor HRQOL following oesophageal cancer surgery. Eligible patients from the United Kingdom will be invited to complete the LASOR tool (Table 1) and the EORTC QLQC30 and OG25 questionnaires. Centres from the existing Association of Upper Gastro-intestinal Surgeons for Great Britain and Ireland (AUGIS) research network will be contacted for inclusion in the study, and patients will be consented locally to participate in the study (Appendix A). Patients will be sent the questionnaire electronically or in paper form based upon their preference indicated at the time of consent to complete the questionnaire at home and avoid reporting bias. Patients who fail to respond or complete the questionnaire will receive one telephone or electronic reminder and if they fail to respond will be excluded from the study.

Measures:

Archival data

This data will be collected retrospectively from the patient medical records (Appendix B).

Demographic information: Data on pre-treatment; patient age, sex, body mass index, ethnic background, socioeconomic status (Carstairs index), education level, smoking status, and medical comorbidities (collated with Charlson co-morbidity index) will be collected through review of medical records.

Tumour and treatment information: Information on stage, tumour location, type of surgery, postoperative complications as defined by the Esophageal Complications

For peer review only

Consensus Group (ECCG) [15], and neoadjuvant and adjuvant therapy used will be collected by reviewing medical records.

Complications after 12months: Specific data on long-term complications including anastomotic stricture requiring dilatation and hiatal hernia, will be collected by reviewing medical records.

Outcome data

Each symptom from the LASOR questionnaire will be graded according to impact upon quality of life and frequency of the symptom, with a composite score from 0 to 5. Each EORTC HROQL symptom item comprised 4 categories on a Likert scale: 1, not at all; 2, a little; 3, quite a bit; 4, very much. The previously developed LASOR symptom tool will be validated against HRQOL as measured by the EORTC QLQC30 and OG25. Patients will also be asked to complete a questionnaire (Appendix C) describing their satisfaction in completing the LASER tool.

Inclusion criteria

- Patients aged over 18 years at the time of surgery.
- Patients treated with oesophagectomy for oesophageal or gastro-oesophageal junctional cancer (Siewert I and II) between January 2015 and June 2018.
- Patients at least 12 months post completion of cancer treatment (surgical or oncological)

Exclusion Criteria

- Any patient who lacks capacity or is unable to provide informed consent.
- Any patient below 18 years of age at the time of surgery.
- Any patient with evidence of cancer recurrence as detected by local centres based upon their own routine follow-up protocol.

For peer review only

Sample size: Using simple nomograms, with a predicted prevalence of poor HRQOL of 45% [14], based upon the previously generated LASOR clinical symptom tool, to validate this tool with a sensitivity and specificity of 80% respectively, a minimum of 640 patients would need to be recruited to the study. We anticipate an expected response rate of 80% and thus 800 patients would need to be recruited to the study.

Statistical methodology:

Each symptom from the LASOR questionnaire will graded according to impact upon quality of life and frequency of the symptom, with a composite score from 0 to 5 (Table 2). Each EORTC HROQL symptom item comprises 4 categories on a Likert scale: 1, not at all; 2, a little; 3, quite a bit; 4, very much. Linear transformation of Likert scores for answers in each conceptual area will be performed as per EORTC recommendations. Symptom scores hence comprise a numeric value from 0 to 100, with higher scores indicating more pervasive symptoms. Poor HRQOL will be defined as having poor function and poor symptom in QLQ-C30 and QlQ-OG25 (by answering “Quite a Bit”/”Very Much” problems to at least one question each in function and symptom scales) [16,17]. Patients who do not answer “Quite a Bit”/”Very Much” will be considered as having good HRQOL. The area under the receiver operating characteristics curve (AUC) will be utilized as a measure of overall accuracy of the prediction model from the LASOR tool in the identification of patients with poor HRQOL as measured by EORTC QLQ-C30 and QlQ-OG25 tools.

For peer review only

Dissemination

Multiple methods of dissemination will be employed to ensure the findings from this research will reach relevant stakeholders including patients, primary care practitioners, scientists, hospital specialists in gastroenterology, oncology and surgery, health policy makers and commissioners as well as healthcare regulatory bodies. The study findings will be presented at international gastroenterology, oncology and surgical research meetings. The findings of this research will also be presented to relevant patient groups. Ultimately, we plan to publish the results of this research in a high impact factor clinical journal to allow widespread dissemination of this research. Further as this trial will be run through the AUGIS research network, the external validity of this tool will be high within the UK population, and thus we anticipate the translation to clinical practice would be faster. Patient acceptability will also be tested and if acceptable, will further facilitate clinical implementation.

Ethics

NHS Health Research Authority **(**North East – York Research Ethics Committee) approval was gained 8th November 2019 (REC reference 19/NE/0352).

Author contributions

SR Markar; Study conception, design, and drafting of full protocol.

EA Griffiths; study design and revising full protocol.

P Behrens; Patient representative involved in study design and revising full protocol.

P Singh; study design and revising full protocol.

For peer review only

R Vohra; study design and revising full protocol.

J Gossage; study design and revising full protocol.

TJ Underwood; study design and revising full protocol.

George B Hanna; study design and revising full protocol

Funding Statement

No funding has been gained for this study at present.

Competing interests

None

Collaborating authors

On behalf of the LASORS study group.

For peer review only

References

1. Fitzmaurice C, Akinyemiju TF, Al Lami FH, et al. Global, regional, national cancer incidence, mortality, years of life lost, years lived with disability, and disability-adjusted life-years for 29 cancer groups, 1990 to 2016: a systematic analysis for the global burden of disease study. JAMA Oncol 2018; 4: 1553 – 68.

2. Siegel RL, Miller KD, Jemal A. Cancer statistics 2018. CA Cancer J Clin 2018; 68: 7–30.

3. Anderson LA, Tavilla A, Brenner H, et al. Survival for oesophageal, stomach and small intestine cancers in Europe 1999–2007: Results from EUROCARE-5. Eur J Cancer 2015; 51: 2144–57.

4. Shapiro J, Van Lanschot JJB, Hulshof MCCM, et al. Neoadjuvant chemoradiotherapy plus surgery versus surgery alone for oesophageal or junctional (CROSS): long-term results of a randomised controlled trial. Lancet Oncol 2015; 16: 1090–8.

5. Alderson D, Cunningham D, Nankivell M, et al. Neoadjuvant cisplatin and fluorouracil versus epirubicin, cisplatin, and capecitabine followed by resection in patients with esophageal adenocarcinoma (UK MRCOE05): an open-label, randomised phase 3 trial. Lancet Oncol 2017; 18: 1249 – 60.

6. Finks JF, Osborne NH, Birkmeyer JD. Trends in hospital volume and operative mortality for high-risk surgery. N Engl J Med 2011; 364: 2128–37.

7. Palser TR, Cromwell DA, Hardwick RH, et al. Re-organisation of oesophago-gastric cancer care in England: progress and remaining challenges. BMC Health Serv Res. 2009; 9:204.

For peer review only

8. Mariette C, Markar SR, Dabakuyo-Yonli TS et al. Hybrid minimally invasive esophagectomy for esophageal cancer. N Engl J Med 2019; 380: 152–62.

9. Bouras G, Markar SR, Burns EM, et al. The psychological impact of symptoms related to esophagogastric cancer resection presenting in primary care: a national linked database study. Eur J Surg Oncol 2017; 43: 454–60.

10. McKernan M, McMillan DC, Anderson JR, et al. The relationship between quality of life (EORTC QLQ-C30) and survival in patients with gastro-oesophageal cancer. Br J Cancer 2008; 98: 888–93.

11. Lagergren P, Fayers P, Conroy T, et al. Clinical and psychometric validation of a questionnaire module, the EORTC QLQ-O25, to assess health-related quality of life in patients with cancer of the oesophagus, the oesophago-gastric junction and the stomach. Eur J Cancer 2007; 43: 2066–73.

12. Noordman BJ, Verdam MGE, Lagarde SM, et al. Effect of neoadjuvant chemoradiotherapy on health-related quality of life in esophageal or junctional cancer: results from the randomized CROSS trial. J Clin Oncol 2018; 36: 268–75.

13. Kauppila JH, Johar A, Lagergren P. Medical and surgical complications and health-related quality of life after esophageal cancer surgery. Ann Surg 2018 [Epub ahead of print].

14. Markar SR, Zaninotto G, Castoro C, et al. LAsting Symptoms after Esophageal Resection (LASER) – European Multi-center Cross-sectional Study. *Presented at International Society for Diseases of Esophagus meeting Vienna 2018.*

15. Low DE, Alderson D, Cecconello I, et al. International consensus on standardization of data collection for complications associated with esophagectomy: esophagectomy complications consensus group (ECCG). Ann Surg 2015; 262: 286–94.

For peer review only

16. Aaronson NK, Ahmedzai S, Bergman B, et al .The European Organisation for Research and Treatment of Cancer QLQ-C30: A quality-of-life instrument for use in international clinical trials in oncology. J Nat Cancer Inst 1993; 85: 365-376.

17. Fayers PM, Aaronson NK, Bjordal K, et al, on behalf of the EORTC Quality of Life Group. *The EORTC QLQ-C30 Scoring Manual (3rd Edition).* Published by: European Organisation for Research and Treatment of Cancer, Brussels 2001.

For peer review only

	**Q1. Do you have any of the following symptoms and how often? Please mark**						**Q2. What is the impact of these symptoms to your quality of life?**		
**Symptom**	**Never**	**Rarely**	**Weekly**	**Daily**	**Multiple times per day**		**None**	**Some**	**Substantial**
Low mood									
Reduced energy or activity tolerance									
Pain on scars from chest									
Heartburn/acid/bile (sour/bitter tasting)									
Diarrhoea (>3 times per day) unrelated to eating									
Bloating or cramping after eating									

Table 1: LASOR symptom tool

For peer review only

Symptom Level	QOL impact and Frequency
**0**	**No symptom present**
**1**	**QOL impact = None**
**2**	**QOL impact = Some & Frequency = rarely / weekly**
**3**	**QOL impact = Some & Frequency = daily / multiple**
**4**	**QOL impact = Substantial & Frequency = rarely / weekly**
**5**	**QOL impact = Substantial & Frequency = daily / multiple**

Table 2: Symptom based grading system including prevalence and impact on Quality of life (QOL) - Each symptom from the LASOR questionnaire will graded according to impact upon quality of life and frequency of the symptom, with a composite score from 0 to 5

Imperial College London

Division of Surgery
Department of Surgery and Cancer
Imperial College London
Biodynamics laboratory, 7th Floor
Charing Cross Hospital
Fulham Palace Road
London W6 8RF
Tel: +44 (0) 203 312 2125
s.markar@imperial.ac.uk
www.imperial.ac.uk

Name of Researchers: Dr Sheraz R. Markar, Professor George B. Hanna, Professor Tim Underwood, Mr Ewen Griffiths, Mr Ravi Vohra, Mr James Gossage, Mr Stuart Mercer, Mr Bilal Alkhaffaf, Mr Arun Ariyarathenam, Mr Shameen Jahoo, Mr John Findlay

Participant Identification Number for this trial:

CONSENT FORM

LAsting Symptoms after Oesophageal Resectional Surgery (LASORS)

1. I confirm that I have read and understood the Patient Information Sheet dated 25.03.19 (version 1) for the above study. I have had the opportunity to consider the information, ask questions and have had these answered satisfactorily. ☐

2. I understand that my participation is voluntary and that I am free to withdraw at any time without giving a reason, and without my medical care, education, employment or legal rights being affected. ☐

3. I agree to my data, including personal identifiable data, being stored on Imperial College London computer system and understand my research data will be stored for 10 years following completion of the study. ☐

4. I understand that sections of my research notes and/ data may be accessed by responsible persons from the research team, from Imperial College London and/or Imperial College Healthcare NHS Trust or from regulatory authorities where it is relevant to the research and in order to check that it is being conducted correctly. ☐

5. I agree for my contact details to be stored on Imperial College London Secure Database in the event that I may request for a research summary. Once the research summary has been sent out, I understand my personal contact details will be deleted. ☐

6. I agree to take part in the above study. ☐

____________________	____________________	____________________
Full name of Participant	Date	Signature
____________________	____________________	____________________
Name of person taking consent	Date	Signature

Consent Form: Version 1.0 Date 25.03.2019
IRAS No: XXX
1 copy to the patient, 1 copy to site file, 1copy to the patient file

For peer review only

For peer review only - http://bmjopen.bmj.com/site/about/guidelines.xhtml

Imperial College London

Division of Surgery
Department of Surgery and Cancer
Imperial College London
Biodynamics laboratory, 7th Floor
Charing Cross Hospital
Fulham Palace Road
London W6 8RF
Tel: +44 (0) 203 312 2125
s.markar@imperial.ac.uk
www.imperial.ac.uk

Name of Researchers: Dr Sheraz R. Markar, Professor George B. Hanna, Professor Tim Underwood, Mr Ewen Griffiths, Mr Ravi Vohra, Mr James Gossage, Mr Stuart Mercer, Mr Bilal Alkhaffaf, Mr Arun Ariyarathenam, Mr Shameen Jahoo, Mr John Findlay

CASE REPORT FORM

LAsting Symptoms after Oesophageal Resectional Surgery (LASORS)

Participant Identification Number for this trial: ....

Name of place of surgery: ………………………………………………………………………….

Current age of patient: ……………………………………………………………………………...

Gender: Male ❍

Female ❍

Height at surgery (cm): ……………………………………………………………………………..

Weight at surgery (kg): ……………………………………………………………………………..

Current weight (kg): ………………………………………………………………………………..

Date of surgery: ……………………………………………………………………………………….

Preoperative medical comorbidties:

- Ischaemic Heart Disease ❍
- Congestive heart failure ❍
- Cerebrovascular disease ❍
- Peripheral Vascular disease ❍
- Dementia ❍
- Chronic obstructive pulmonary disease ❍
- Peptic ulcer disease ❍
- Mild liver disease ❍
- Diabetes ❍
- Renal disease ❍

ASA Grade:

- I ❍
- II ❍
- III ❍
- IV ❍

For peer review only - http://bmjopen.bmj.com/site/about/guidelines.xhtml

For peer review only

Anastomosis location: Cervical ❍

Thoracic ❍

Surgical technique: Transhiatal ❍

Ivor-Lewis (2-stage) ❍

McKeown (3-stage) ❍

Left thoracoabdominal ❍

Colonic interposition ❍

Other – please specify ❍

[ ]

How was the anastomosis constructed?

Linear Stapler ❍

Circular Stapler (please state diameter if known ❍

Hand sewn ❍

Alignment of anastomosis: End to end ❍

End to side ❍

Side to side ❍

Other (please specify) ❍

[ ]

Surgical access:

Total Minimally Invasive Oesophagectomy ❍

Hybrid Minimally Invasive Oesophagectomy (Open abdominal and thoracoscopy) ❍

Hybrid Minimally Invasive Oesophagectomy (Laparoscopy and open thoracotomy) ❍

Total Open Oesophagectomy ❍

Pyloric management:

No drainage procedure ❍

Pyloroplasty ❍

Pyloromyotomy ❍

Manual pyloric dilatation or stretch ❍

Botulinum injection ❍

Other (please specify) ❍

[ ]

For peer review only

For peer review only - http://bmjopen.bmj.com/site/about/guidelines.xhtml

Was a feeding jejunostomy inserted? Yes ❍
No ❍

Neoadjuvant therapy: Combination chemoradiotherapy ❍
Radiotherapy alone ❍
Chemotherapy alone ❍
None ❍

Chemotherapy or chemoradiotherapy regimen:

CROSS ❍
MAGIC ❍
FLOT ❍
Other ❍
Unknown ❍

CROSS - Paclitaxel (50 mg/m2) and Carboplatin (Area-Under-Curve = 2) administered by i.v. infusion on days 1, 8, 15, 22, and 29. External beam radiation with a total dose of 41.4 Gy given in 23 fractions of 1.8 Gy, 5 fractions a week.

MAGIC - Three preoperative and three postoperative cycles of intravenous epirubicin (50 mg per square meter of body-surface area) and cisplatin (60 mg per square meter) on day 1, and a continuous intravenous infusion of fluorouracil (200 mg per square meter per day) for 21 days.

Adjuvant therapy: Combination chemoradiotherapy ❍
Radiotherapy alone ❍
Chemotherapy alone ❍
None ❍

Pathological T stage: T0 ❍
T1 ❍
T2 ❍
T3 ❍
T4 ❍

Pathological N stage 7th Edition: N0 ❍
N1 ❍
N2+ ❍

Pathological TNM stage 7th Edition: Stage I ❍
Stage II ❍
Stage III ❍
Stage IV ❍

For peer review only

For peer review only - http://bmjopen.bmj.com/site/about/guidelines.xhtml

Tumour histological subtype: Adenocarcinoma ❍

SCC ❍

Other ❍

Postoperative complications (use complications platform from Low DE et al. Annals of Surgery 262 (2):286-294 2015):

- Pulmonary ❍
- Cardiac ❍
- Gastrointestinal ❍
- Urologic ❍
- Thromboembolic ❍
- Neurologic/psychiatric ❍
- Infection ❍
- Wound/diaphragm ❍
- Other ❍

For peer review only

Please specify pulmonary complication:

- Pneumonia ❍
- Pleural effusion requiring additional drainage procedure ❍
- Pneumothorax requiring treatment ❍
- Atelectasis mucous plugging requiring bronchoscopy ❍
- Respiratory failure requiring reintubation ❍
- Acute respiratory distress syndrome (Berlin definition) ❍
- Acute aspiration ❍
- Tracheobronchial injury ❍
- Chest tube maintenance for air leak for >10 days postoperatively ❍

Please specify cardiac complication:

- Cardiac arrest requiring CPR ❍
- Myocardial infarction as defined by the World Health Organisation ❍
- Dysrhythmia atrial requiring treatment ❍
- Dysrhythmia ventricular requiring treatment ❍
- Congestive heart failure requiring treatment ❍
- Pericarditis requiring treatment ❍

Please specify gastrointestinal complication:

- Oesophagoenteric leak from anastomosis, stapled line or localised conduit necrosis ❍
- Conduit necrosis/failure ❍
- Ileus defined as small bowel dysfunction preventing or delayed enteral feeding ❍
- Small bowel obstruction ❍
- Feeding J-tube complication ❍

For peer review only - http://bmjopen.bmj.com/site/about/guidelines.xhtml

Pyloromyotomy/pyloroplasty complication ❍

Clostridium Difficile infection ❍

Gastrointestinal bleeding requiring intervention or transfusion ❍

Delayed conduit emptying requiring intervention or delayed discharge or requiring maintenance of nasogastric drainage >7 days postoperatively ❍

Pancreatitis ❍

Liver dysfunction ❍

Please specify urological complication:

Acute renal insufficiency (defined as doubling of baseline creatinine) ❍

Acute renal failure requiring dialysis ❍

Urinary tract infection ❍

Urinary retention requiring reinsertion of urinary catheter, delaying discharge or discharge with a urinary catheter ❍

Please specify thromboembolic complication:

Deep vein thrombosis ❍

Pulmonary embolus ❍

Stroke (CVA) ❍

Peripheral thrombophlebitis ❍

Please specify neurologic/psychiatric complication:

Recurrent nerve injury ❍

Other neurologic injury ❍

Acute delirium ❍

Delirium tremens ❍

Please specify infection complication:

Wound infection requiring opening of wound or antibiotics ❍

Central i.v. line infection requiring removal of antibiotics ❍

Intrathoracic/intra-abdominal abscess ❍

Generalised sepsis ❍

Other infections requiring antibiotics ❍

Please specify wound/diaphragm complication:

Thoracic wound dehiscence ❍

Acute abdominal wall dehiscence/hernia ❍

Acute diaphragmatic hernia ❍

Please specify other complication:

For peer review only
For peer review only - http://bmjopen.bmj.com/site/about/guidelines.xhtml

Chyle leak ❍

Reoperation for reasons other than bleeding, anastomotic leak or conduit necrosis ❍

Multiple organ dysfunction syndrome ❍

Late symptoms requiring intervention (>90 days following surgery):

Stricture ❍

Delaying emptying (requiring dilatation) ❍

Other ❍

Maximum Clavien-Dindo Classification of postoperative complication (include 30-day complications or in-patient stay – whichever is the longest)

I ❍

II ❍

III ❍

IV ❍

For peer review only

For peer review only - http://bmjopen.bmj.com/site/about/guidelines.xhtml

LASORS tool - Satisfaction

INSTRUCTIONS:

- Please try to answer all questions.
- Circle the answer that you believe describes you best.

1	2	3	4	5	6	7
Disagree Completely			Neutral			Agree Completely

1. LASOR questionnaire was easy to complete.	1	2	3	4	5	6	7
2. LASOR questionnaire was understandable.	1	2	3	4	5	6	7
3. LASOR questionnaire did not take much time to complete.	1	2	3	4	5	6	7
4. LASOR questionnaire included the main symptoms I experience.	1	2	3	4	5	6	7
5. I preferred the LASOR questionnaire to the EORTC questionnaires	1	2	3	4	5	6	7
6. I would recommend using the LASOR questionnaire to other oesophageal cancer survivors.	1	2	3	4	5	6	7
